# Design and characterization of an 87k SNP genotyping array for Arctic charr (*Salvelinus alpinus*)

**DOI:** 10.1371/journal.pone.0215008

**Published:** 2019-04-05

**Authors:** Cameron M. Nugent, Jong S. Leong, Kris A. Christensen, Eric B. Rondeau, Matthew K. Brachmann, Anne A. Easton, Christine L. Ouellet-Fagg, Michelle T. T. Crown, William S. Davidson, Ben F. Koop, Roy G. Danzmann, Moira M. Ferguson

**Affiliations:** 1 University of Guelph, Department of Integrative Biology, Guelph, Ontario, Canada; 2 University of Victoria, Department of Biology, Victoria, British Columbia, Canada; 3 Fisheries and Oceans Canada, Centre for Aquaculture and Environmental Research, West Vancouver, British Columbia, Canada; 4 Simon Fraser University, Molecular Biology and Biochemistry, Burnaby, British Columbia, Canada; Swansea University, UNITED KINGDOM

## Abstract

We have generated a high-density, high-throughput genotyping array for characterizing genome-wide variation in Arctic charr (*Salvelinus alpinus*). Novel single nucleotide polymorphisms (SNPs) were identified in charr from the Fraser, Nauyuk and Tree River aquaculture strains, which originated from northern Canada and fish from Iceland using high coverage sequencing, reduced representation sequencing and RNA-seq datasets. The array was designed to capture genome-wide variation from a diverse suite of Arctic charr populations. Cross validation of SNPs from various sources and comparison with previously published Arctic charr SNP data provided a set of candidate SNPs that generalize across populations. Further candidate SNPs were identified based on minor allele frequency, association with RNA transcripts, even spacing across intergenic regions and association with the sex determining (*sdY*) gene. The performance of the 86,503 SNP array was assessed by genotyping Fraser, Nauyuk and Tree River strain individuals, as well as wild Icelandic Arctic charr. Overall, 63,060 of the SNPs were polymorphic within at least one group and 36.8% were unique to one of the four groups, suggesting that the array design allows for characterization of both within and across population genetic diversity. The concordance between *sdY* markers and known phenotypic sex indicated that the array can accurately determine the sex of individuals based on genotype alone. The Salp87k genotyping array provides researchers and breeders the opportunity to analyze genetic variation in Arctic charr at a more detailed level than previously possible.

## Introduction

Arctic charr (*Salvelinus alpinus*) has a Holarctic distribution spanning marine and freshwater ecosystems and is one of the most morphologically and ecologically diverse vertebrates [1,2]. The species is subdivided into several genetically differentiated phylogeographic groups, which are thought to have diverged in refugia during the early to mid-Pleistocene [1,3]. Arctic charr are of economic importance and are an attractive option for the expansion of aquaculture production at northern latitudes [4]. Characteristics such as early maturation, poor salinity tolerance and uneven growth limit current Arctic charr aquaculture production [5,6]. Improving the characterization of the Arctic charr genome will allow for detailed study of the genetic basis of these important traits and provide a starting point for selective breeding programs that aim to improve economically important aspects of the Arctic charr phenotype using genomic information.

Studies of the genetic architecture of traits and the discovery of quantitative trait loci (QTL) in Arctic charr have been limited by the relatively small numbers of available genetic markers [7–12]. Low cost methods for massively parallel genetic marker discovery through reduced representation sequencing [13,14] have resulted in the discovery of thousands of novel single nucleotide polymorphisms (SNPs) in Arctic charr and led to the creation of a 4,508 marker genetic linkage map for the Canadian Fraser strain [15]. The linkage map has been used to characterize the evolutionary history of Arctic charr chromosomes and identify homologous chromosomal regions in closely related salmonid species. A large suite of SNPs has also been identified through a transcriptomic analysis of salinity tolerance [16]. In addition to these genomic resources, a recently developed Arctic charr reference genome assembly and transcriptome annotation [17] have allowed for the identification of orthologous genes between Arctic charr, other salmonids and northern pike (*Esox lucius*) that might provide insight on the adaptive divergence of salmonid species.

Further insights into the genetics and evolution of Arctic charr require a high-throughput, high-density genotyping array so that fish can be genotyped for a large number of markers in a cost-effective manner. High-density SNP genotyping assays (6K to 285K) for other salmonids such as rainbow trout (*Oncorhynchus mykiss*) and Atlantic salmon (*Salmo salar*) [18–22] have been used successfully to determine the genetic basis of growth, maturation and disease resistance traits [23–28] and to characterize population structure [29,30]. Genotyping arrays have also been designed for other aquaculture species such as carp (*Cyprinus carpio*) [31] and some have also been designed to work on multiple species, such as those for Pacific and European oysters (*Crassostrea gigas* and *Ostrea edulis*) [32] and blue catfish and channel catfish (*Ictalurus furcatus* and *I*. *punctatus*) [33].

The creation of SNP arrays for aquaculture species follow previous developments in terrestrial livestock (such as poultry and cattle) and data from these arrays are now being successfully applied in genomic selection programs that improve the performance of aquaculture populations for important traits such as disease resistance [34–40]. Within Atlantic salmon and rainbow trout, GWAS based on genotyping array data have successfully identified QTL for important aquaculture traits such as fillet yield, growth and body mass and for Atlantic salmon also identified a single locus (*vgll3*) that controls variation in age at maturity [24–26,41–43]. Characterizing genome-wide variation within and across populations of Arctic charr using a genotyping array would pave the way for genome-wide association analyses (GWAS) and identification of the genetic basis of important aquaculture traits. Pairing accurate genotype information obtained from an array with knowledge of the Arctic charr genome [17] could also provide fundamental information about the distribution and evolution of functional genes as well as insights into differences in genomic architecture between Arctic charr and its close taxonomic relatives.

Our aims were to: (1) Expand the number of SNPs identified in Arctic charr; (2) Determine the position of SNPs within the genome identified through different molecular approaches for comparative analysis; (3) Design a SNP genotyping array that captures the diversity of Arctic charr by incorporating SNPs identified in a diverse suite of populations and (4) Design an array that contains SNPs located in functional genes and coverage of intergenic regions through the inclusion of markers that are evenly spaced throughout the genome. Putative SNPs were identified in fish from the three major Canadian aquaculture strains (Fraser, Nauyuk and Tree River) that were founded from populations in northern Canada [44] as well as Icelandic fish originating from two lakes (Þingvallavatn and Vatnshlíðarvatn) and populations in or near Lake Mývatn. Following the creation of the genotyping array, we tested its performance with samples from the same populations/strains used for SNP discovery as well as fish from additional Icelandic populations (lakes Galtaból, Mjóavatn, Mývatn, and Svínavatn; Fljótaá River) to discover the number of polymorphic array markers in the different groups. By designing the array using markers identified in different groups, we hoped to create a tool that could characterize genetic variation across the range of the species.

## Materials and methods

### Ethics statement

Animals were reared and sampled in compliance with the animal utilization protocols (AUP) #3174 and #2431, which were approved by the University of Guelph Animal Care Committee.

### Sample information

The fish used for SNP discovery and testing of the array originated from aquaculture strains in Canada and natural populations in Iceland (Table 1). The Nauyuk and Tree River aquaculture strains were founded from adults obtained in the 1970’s and 1980’s from locations of the same name in Nunavut, Canada while the founders of the Fraser strain were collected from the Fraser River, Labrador Canada between 1980 and 1984 [44,45]. The Tree River and Nauyuk adults and families (pure strain and hybrids) used in the current study were obtained from Icy Waters, Ltd (Whitehorse, Yukon, Canada) while those from the Fraser strain were obtained from the Alma Aquaculture Research Station (Alma, Ontario, Canada) and the Coastal Zones Research Institute (CZRI) (Shippagan, New Brunswick, Canada). SNP discovery in the Icelandic fish was based on eight full-sib families produced from adults collected from the lakes Þingvallavatn and Vatnshlíðarvatn (see Parsons *et al*. 2011 [46] for details) and fish sampled from Lake Mývatn and 11 nearby lava caves. The array was tested on Icelandic fish sampled from six lakes (Galtaból, Mývatn, Mjóavatn, Svínavatn, Þingvallavatn and Vatnshlíðarvatn), a river (Fljótaá) and lava caves near Lake Mývatn. The Nauyuk and Tree River populations are part of the Arctic phylogeographic group, while the Fraser strain and Icelandic charr are part of the Atlantic phylogeographic group [1].

**Table 1:** Sources of fish, sequence data types used in the design and testing of the Arctic charr genotyping array.

Source of fish	Methodology	Individuals	Structure	Publication
Fraser aquaculture strain (Coastal Zones Research Institute, New Brunswick, Canada)	GBS	91	Full sib family	Nugent *et al*. 2017
	mRNA-seq	18	Two full sib families	Norman *et al*. 2014
Fraser aquaculture strain (Alma Aquaculture Research Station)	GBS	108	Two half sib families	
	Array testing	33*	Population	
Nauyuk aquaculture strain (Icy Waters, Yukon, Canada)	GBS	24	Population	
	Array testing	42^†^	Population	
Nauyuk–Tree River aquaculture strain hybrids (Icy Waters)	RAD-seq	238	Nine hybrid Families	Christensen *et al*. 2018
Fraser–Nauyuk aquaculture strain hybrids	RAD-seq	67	Two hybrid families	Christensen et al. 2018
Nauyuk–Tree River aquaculture strain hybrids	High coverage sequencing	8	Population	Christensen *et al*. 2018
Tree River aquaculture strain (Icy Waters)	Array testing	18	Population	
Lake Þingvallavatn Iceland	GBS	320	Four full sib families	Parsons *et al*. 2011
	Array testing	95	Population	
Lake Vatnshlíðarvatn, Iceland	GBS	362	Four full sib families	Parsons *et al*. 2011
	Array testing	64	Population	
Lake Mývatn, Iceland	GBS	9	Population	
Mývatn lava caves, Iceland	GBS	39	Population	
	Array testing	20^⧧^	Population	
Lake Galtaból, Iceland	Array testing	57	Population	
Lake Mjóavatn, Iceland	Array testing	31	Population	
Lake Svínavatn, Iceland	Array testing	90	Population	
River Fljótaá, Iceland	Array testing	32	Population	

* Three of the Fraser strain individuals in the test set were also parents of the two half sib families utilized in GBS in SNP discovery.

^†^ Twenty-four of the Nauyuk strain individuals in the test set were also used in SNP discovery.

^⧧^All 20 of the Mývatn lava cave fish in the test set were also used for SNP discovery

### SNP discovery

Candidate SNPs for the array were detected using a variety of sequencing methodologies. First, genotype-by-sequencing (GBS) [14] was performed on 951 individuals from multiple sources (Table 1). DNA was extracted from tissue using a commercial kit (Qiagen DNeasy Blood & Tissue) as per the manufacturer’s instructions. Samples were quantified using a Qubit Fluorometer and diluted to a concentration of 75ng/uL. For each individual, 30μl of sample was digested with the restriction enzyme EcoT22I and unique barcode adapters were ligated to the restriction cut sites. After unique barcodes were added, sequencing primers and the DNA samples from all individuals were pooled and amplified through the polymerase chain reaction (PCR) and sequenced (see Nugent *et al*. 2017 [15] for details).

After sequencing, raw fastq files were filtered for quality control in Trimmomatic using default parameters [47] (Version: Trimmomatic-0.36). Following quality control, data were analyzed using the software package Stacks for *de novo* SNP identification [48] (Version: 1.44). The subprograms of Stacks were implemented sequentially (process_radtags, ustacks, cstacks, sstacks using default parameters). For the Fraser and Icelandic families (Table 1), the inheritance of alleles could be tracked, so the Stacks ‘genotypes’ module was used to generate output information on SNP variation. The Stacks ‘populations’ module was used to generate genotype output data for the population samples (Nauyuk and Mývatn area), where the relationships of individuals were unknown.

The GBS dataset was processed with Stacks twice, the first time using a process_radtags trim parameter (-t) of 85 and the second time using a trim parameter of 40 (-t 40). This dual approach was used because a trim parameter of 85 caused stacks to eliminate any reads shorter than 85 bp in length. Previous analysis of GBS data in the production of the first generation Arctic charr SNP linkage map (NCBI sequence read archive (www.ncbi.nlm.nih.gov/sra) BioProject accession number #SRP026259 and BioSample accession numbers #SAMN06165956 and #SAMN06165957) [15] identified SNPs on sequences shorter than 85bp in length. Therefore, the lower cutoff threshold (40bp) was used to retain shorter reads in an attempt to observe the short read SNPs in newly sequenced individuals. To prevent redundancy, SNPs with identical polymorphisms and base pair sequences from the two Stacks analyses and the first generation linkage map [15] were identified and a single copy was retained.

SNPs were filtered in different ways depending on the source. Those derived from families were analyzed manually to remove SNPs that met one of the following criteria in all families: 1. >50% of progeny with missing genotypes; 2. detection of erroneous genotypes (e.g., presence of bb genotypes when parents had aa and ab genotypes); and 3. significant segregation distortion (analyzed in the linkmfex_V3 program ‘OneMap_Segregation_Distortion_Check’) [49]. Markers derived from population samples (Nauyuk and Mývatn area) were filtered to retain SNPs with observed minor allele frequencies > = 0.05. Finally, SNPs meeting the above criteria were retained only if the short DNA sequences [40–85 bp in length) containing the SNP aligned to a single location in the Arctic charr draft genome, as determined through a Burrows-Wheeler alignment (NextGene, SoftGenetics LLC). SNPs sequences that did not align or aligned to two or more locations were omitted.

Second, 11 families (nine Nauyuk x Tree River and two Fraser x Nauyuk hybrid families, Table 1 of Christensen *et al*. 2018 [17]) were RAD-sequenced (Methods section: ‘Data processing and genetic map construction’ in Christensen *et al*. 2018 [17]). SNPs that passed all quality control steps were used to construct a genetic linkage map and were added to the list of candidate markers.

Third, eight hybrid fish derived from crosses between the Tree River and Nauyuk strains were each sequenced on one lane of an Illumina HiSeq2500 (~40x coverage, paired-end sequencing) [17]. A Burrows-Wheeler alignment was performed to align raw paired-end reads (no filtering or trimming applied) to the Arctic charr draft genome. Within the program SAMtools, the Mpileup function was used with Bcftools to generate SNPs from the alignment data. SNPs were filtered based on the following criteria: filter = ‘.’, quality score for alternate assertion ≥ 20, RMS mapping quality ≥ 30, genotype quality ≥ 20, 1 ≤ depth ≤ 100. SNPs remaining after filtering (Table 6 in Christensen *et al*. 2018 [17]) were retained for the current analysis.

Fourth, SNPs were identified from a previous transcriptomic analysis of Fraser strain Arctic charr [16]. These SNPs were initially characterized during a *de novo* assembly that was performed using mRNA sequence libraries from 18 individuals. Briefly transcriptome assemblies were constructed in the Velvet-Oases software package using eight different k-mer lengths (33, 41, 49, 57, 65, 73, 81, 89) [50,51]. Contigs less than 300bp in length were removed and the assemblies were merged using the Oases-M module and a k-mer length of 105. CD-HT-EST [52] was used to cluster contigs where shorter sequences shared 95% identity within local alignments to larger sequences. SNPs were then retained only if the contig containing the SNP aligned to a single location in the Arctic charr draft genome, as determined through a Burrows-Wheeler alignment (NextGene, SoftGenetics LLC).

### Selection of SNPs for the genotyping array

We first selected SNPs that had been detected by more than one sequencing platform (i.e., high coverage, GBS, RAD-seq, RNA-seq). These were considered as cross validated if SNPs in the two datasets were found at the same base pair position in the Arctic charr draft genome (precursor to the newest Arctic charr genome build, GenBank accession: GCA_002910315.2) [17] and if they had matching alleles. We used the draft genome as a reference during array design as the genome build was incomplete at the time. We next prioritized SNPs identified through GBS that met one of the following criteria: (a) SNP was detected in two populations, (b) SNP had a minor allele frequency >0.05 in a population, (c) SNP was segregating in two or more families. We filtered out most SNPs with G/C and A/T polymorphisms because these require twice as many assays on Affymetrix arrays and are therefore inefficient. However, we retained those in the Icelandic samples to maximize the number of polymorphisms observed in these individuals.

We next included markers that could be used to determine the genotypic sex of individuals. The eight libraries from the high coverage sequencing SNP data were compared to a partial transcript for the Arctic charr sex-determining gene, *sdY* (GenBank accession: JF826022.1), using Burrows-Wheeler Aligner (BWA) [53]. SAMtools Mpileup was used to call SNPs using the results from these BWA alignments [54].

We next focused on SNPs identified in the eight Nauyuk x Tree River hybrid individuals subjected to high coverage sequencing that had not been selected through cross validation. These SNPs were placed in a MySQL database and filtered based on the following initial parameters: depth (5 ≤ DP ≤ 45), quality score (QUAL ≥ 20), genotype quality (GQ ≥ 20) and mapping quality (MQ ≥ 30). In order to identify which SNPs fell within transcripts, Blastn was used to compare 101bp probes (SNP at bp 51) to the transcriptome from Christensen *et al*. [17] and SNPs were labeled based on their presence or absence within transcripts. The SNPs were compared to the transcriptome and not directly to the reference genome because the reference genome had not been finalized at the time of this analysis. SNPs from different contigs aligning to the same transcript were excluded due to the potential ambiguity. SNPs were then excluded if they had less than 35bp of flanking sequence on either side. A/T and G/C variants were filtered from the dataset and SNPs were split into rare (0.05 ≤ AF < 0.15) and common SNPs (0.15 ≤ AF ≤ 0.85). A set of rare intergenic and intragenic SNPs were selected to produce ~900Kb intervals between markers. Additionally, common SNPs not found in transcripts were selected to produce a set of common intergenic markers spaced at ~62Kb intervals. Finally, to fill the remaining room on the array, SNPs from the GBS, and RNA-seq datasets that had been successfully aligned to the draft genome but that were rejected in previous filtering steps due to a lack of available information (no minor allele frequencies or segregation data) or unsuccessful cross validation were included in the initial selection of SNPs. These SNPs were given the lowest priority in array design due to their lack of validation and putative nature.

Following the initial selection, 103,932 candidate SNPs were submitted to Affymetrix for review in 71-mer format, with both alleles for the SNP on the forward strand provided at base pair position 36. *In silico* analysis produced a probability of conversion to a reliable assay for each SNP (p-convert score). This returned a set of 80,786 SNPs (77.7%) from the initial submission with a ‘recommended’ or ‘neutral’ designation. To fill the remaining spots on the array, 13,912 additional intergenic common SNPs from the high coverage sequencing dataset were added and the revised set of candidate markers was resubmitted. After resubmission to Affymetrix for array tiling, the Salp87k array design with 86,503 SNPs was finalized (S1 File).

Following design and construction of the Arctic charr genotyping array, an additional Blast alignment was conducted to align the array SNPs to the final Arctic charr reference genome assembly (GenBank assembly accession: GCA_002910315.2) [17]. We also determined how well the Salp87k array was representing the genes within the genome. The positions of SNPs in the Arctic charr reference genome were compared to location of the 42,439 genes reported in genome annotation file (GenBank assembly accession: GCA_002910315.2) in order to count the number of genes that contained an array marker between their base pair start and end positions.

### Testing of the genotyping array

To investigate the ability of the array to characterize the genetic diversity of divergent populations, SNP variation in a test set of 482 individuals including fish from the four groups (three aquaculture strains and wild fish from Iceland) was evaluated (Table 1). Three of the Fraser fish, 24 of the Nauyuk fish and 20 fish from the caves near Lake Mývatn in the test set were previously used for SNP discovery with GBS.

Aliquots of DNA were sent to the Clinical Genomics Centre at Mt. Sinai Hospital, Toronto, Canada and genotyped as per the manufacturer’s instructions. Genotypic data were imported into the Axiom Analysis Suite (Version 3.1.51) and filtered following the manufacturer’s ‘best practices workflow’ (diploid genome, filtered for dish quality control values >0.82, quality control call rate > 0.97 and average call rate for passing samples > 0.98). Genotypic data for the four groups were generated in separate Axiom Analysis Suite sessions, following the manufacturer’s ‘best practices workflow’. A recommended SNP was one whose genotype data met all quality control thresholds (Axiom^™^ Analysis Suite User Manual version 3.1). Recommended SNPs for each group were obtained and compared to one another to assess the number of assays that were polymorphic (and therefore informative) within the different groups. Finally, we validated the ability of the *sdY* associated markers to identify sex by comparing genotypes to phenotypic sex based on visual examination of the gonads in 446 of the test fish.

## Results and discussion

### SNP discovery and selection

Cross validation of SNPs between sequencing platforms and the filtering of GBS data produced a set of 19,587 SNPs that were given the highest priority in array design. Of these, 14,768 SNPs were cross validated between the high coverage sequencing and one of the smaller data sets (GBS, RAD-seq, RNA-seq) (Table 2). We detected no overlap in SNP identity among the smaller data sets. This is partially due to lower genome coverage and the use of different restriction enzymes in the two reduced representation sequencing data sets. Of the GBS-derived SNPs, 4,276 were cross validated between families from two or more populations but 1,733 of these had already been identified through cross platform validation leaving 2,443 for addition to the high priority list. The population samples subjected to GBS (Table 1) yielded 1,171 additional SNPs based on observed minor allele frequencies (>0.05 in at least one population). The remaining 1,205 SNPs were selected because they were observed in at least two Fraser strain families. Of the GBS-derived SNPs, 1,741 were omitted due to being A/T or G/C variants, leaving a final set of 17,846 high priority markers for array design.

**Table 2:** Summary of the number of candidate SNPs derived from each data source and the number of SNPs from each data source that were included in the final array design.

Data Source	Number included on array	Candidates for array design	Conversion rate
**High coverage sequencing & GBS cross validation**	3,149	5,451	57.8%
**High coverage sequencing & RNA-seq cross validation**	368	583	63.1%
**High coverage sequencing & RAD-seq cross validation**	3,875	8,734	44.36%
**GBS**	6,046	14,959	40.5%
**RNA-seq**	10,491	14,922	70.3%
**High coverage sequencing**	62,568	59,277 + 13,912*	85.5%
**sdY markers**	6	6	100%
**Totals**	86,503	117,844*	73.4%

*The initial candidate list was 103,932 SNPs. When this initial set of candidates failed to yield enough SNPs recommended for use on the array 13,912 high coverage SNPs were added to the list of candidates

In addition to the 17,846 high priority markers, the initial candidate marker set included SNPs from the high coverage sequencing data (59,277), the sex associated markers (6) and non-cross validated markers from the GBS and RNA-seq datasets (26,803) for a total of 103,932 markers. The high coverage sequencing dataset yielded the largest set of SNPs in the initial submission but was constructed using sequence data from only eight individuals. This meant that there was relatively low-resolution allele frequency information available to inform decisions about which markers to include. Care was taken to assess the genomic location of the SNPs from these eight individuals and to select SNPs that represented as many genes as possible and also provide even coverage of intergenic regions. We aimed to directly represent as many genes as possible on the array so that future analyses utilizing the array, such as genome-wide association studies, could accurately identify potential causative genes associated with important SNPs. The lack of validation of most of the SNPs from the eight individuals means that we cannot rule out the possibility that the observed polymorphisms could be the result of sequencing error or other non-biological causes. Thus, these were considered putative in nature prior to validation through assessment of array performance.

The different data sources utilized in SNP discovery were complimentary, providing detail on marker frequency and segregation in populations (GBS, RAD-seq) or high depth of coverage and genomic context (RNA-seq, high coverage sequencing) (Table 2). SNPs from the reduced representation sequencing methods had the highest quality supporting information (allele frequencies, observed segregation) but were the least abundant data source. Alternatively, the high coverage sequencing data had a large library of SNPs to select from, but the supporting information was scant (allele frequencies based on just 8 individuals). By using SNPs from these different data sources, we were able to select the best candidates for array design and give them the highest priority for inclusion on the array.

### Genotyping array performance

For each of the four groups (Fraser strain, Nauyuk strain, Tree River strain, Icelandic), more than 62,000 SNPs were recommended for use by the Axiom Analysis Suite and identified as either monomorphic or polymorphic (Table 3). It is important to note that different subsets of the markers on the array were recommended for use within the different groups. In total, 79,692 of the SNPs on the array were recommended for use within at least one of the four groups. Possible reasons for a SNP not being recommended for use in none of the groups include: the existence of off target SNP variants in the analyzed individuals, poor SNP call rates, or other sequence differences between the array probe set for the given SNP and the DNA sequence of the individuals being genotyped. These issues can be strain-specific, therefore causing certain markers to be recommended for use within Arctic charr derived from one strain and not recommended for individuals of a different strain.

**Table 3:** The number of polymorphic and monomorphic SNPs observed within the different test groups of Arctic charr. The number of polymorphisms unique to each strain (Unique to strain) and the number shared with at least one other strain (Multiple strains) are shown. ‘Total recommended’ indicates markers classified as ‘recommended’ by the Axiom Analysis Suite’s best practices workflow. Polymorphic markers include the Axiom Analysis Suite, ‘polymorphic high resolution’ and ‘no minor homozygote’. Across the four groups, 79,692 unique SNPs were recommended for use.

		Fraser	Nauyuk	Tree River	Icelandic
**Polymorphic**	Unique to strain	1,864 (9.4%)	10,924 (24.2%)	8,551 (22.3%)	1,864 (13.1%)
	Multiple strains	17,898	34,250	29,865	12,329
	Total observed	19,762	45,174	38,416	14,193
**Monomorphic**		42,898	25,151	25,531	54,602
**Total Recommended**		62,660	70,325	63,947	68,795

For each group, between 14,000 and 46,000 polymorphic markers were identified. The highest number of polymorphic markers was observed in the Nauyuk strain (45,174; 64.2% of the recommended SNPs), while the lowest number of SNPs was seen in the Icelandic fish (14,193; 20.6%), despite the larger number of fish genotyped. This pattern was expected, as a large number of the SNPs included in the design of the array were identified from the Nauyuk and Tree River strains as the result of high coverage sequencing. The discovery and selection of SNPs for inclusion on the array could have been improved and the utility of the array maximized by analyzing all of the population samples with the high coverage sequencing method. However, it appears that the array is still able to characterize variation within Fraser and Icelandic fish, albeit to a lesser extent. Reduced representation sequencing, although yielding fewer markers in the Fraser and Icelandic fish, did provide more representative estimates of minor allele frequency and other metrics of SNP efficacy than the high coverage sequencing dataset.

### Population specificity

**Fig 1:**
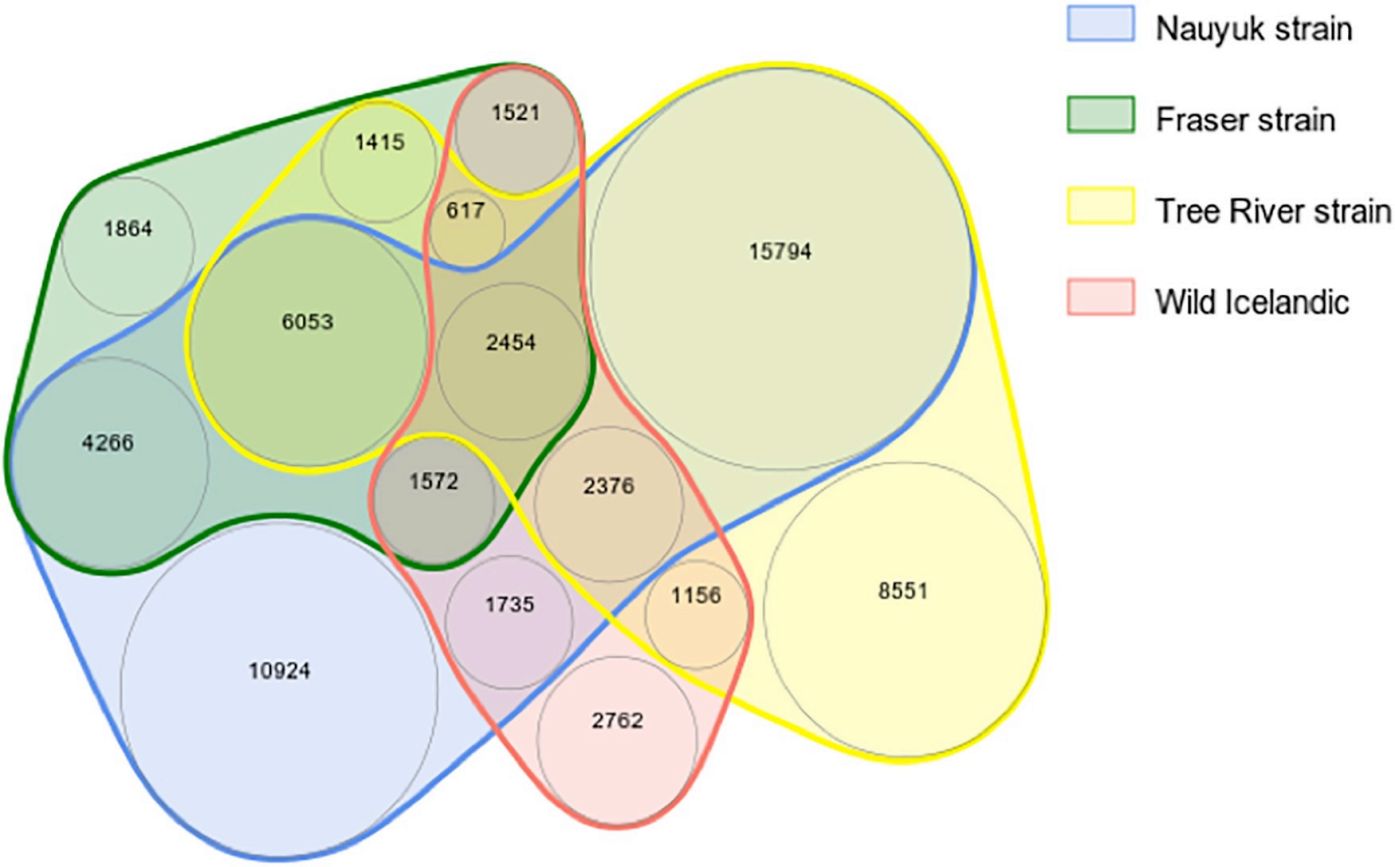
A Venn diagram depicting the number of unique and shared polymorphic SNPs across the four groups of Arctic charr in the test set. In total, 63,060 of the 86,503 SNPs on the Salp87k genotyping were identified as polymorphic while 21,785 were polymorphic within only one of the groups.

A total of 63,060 polymorphic markers were observed (72.9% of the markers on the array) across the four populations of Arctic charr in the test set (Fig 1, Table 3, S3 File). Of the total, 36.8% (22,203) were polymorphic within only one of the four groups, while 63.2% were polymorphic in multiple groups. This suggests that the Salp87k genotyping array is an effective tool for characterizing genetic variation within populations as well as for differentiation among populations. The 23,440 array SNPs that were not verified as informative within any of the four groups may include some SNPs that are not true biological polymorphisms. As more individuals are genotyped with the array, we will be able to better characterize the number of true SNPs on the array, as well as the number of putative SNPs that were included in the final design that fail to yield biologically relevant information in any circumstances.

The percentage of SNPs shared between groups appeared to be a function of geographic separation rather than phylogeographic grouping (S3 File). The two populations in the closest proximity (from the same phylogeogaphic group) showed the greatest percentage of shared markers by far. Of the 56,913 total unique polymorphic markers from the Nauyuk and Tree River populations, 46.9% of SNPs (26,677) were polymorphic within both groups. The two groups derived from the Atlantic phylogeographic group (Fraser and Icelandic) had a lower percentage of shared SNPs (22.2%), which was similar to that between the Fraser and Tree River strains even though they belong to different phylogeographic groups.

The array is likely to be of value for the study of cultured and wild populations of Arctic charr in Canada and Iceland. Results of the test set showcase the ability of the array to characterize genetic variation in the three major Canadian aquaculture strains for use in selective breeding programs. Moreover, since these strains were founded relatively recently (1974–1988), it is possible that the array could be effective at characterizing genetic variation in wild Canadian populations. However, array performance in wild fish would need to be tested given that the aquaculture strains were created with small numbers of founders [44,45] and therefore may not be genetically representative of wild populations. The array was also able to capture genetic variation in the Icelandic populations studied but less optimally. Given that less genetic information was available from Icelandic individuals during the design of the array, SNPs from Icelandic individuals were prioritized in an effort to optimize performance in the genotyping of these fish. Even though the numbers of Icelandic test individuals far outnumbered those from the three Canadian aquaculture strains, they had the lowest number of observed polymorphic SNPs (~15 K). However, this number is suitable for many population genetic/genomic applications but would be less optimal for fine scale genomic analyses. Thus, it may be necessary to develop a location specific array, similar to what has occurred in Atlantic salmon [18–20] for certain applications such as genomic selection.

### Genome coverage

**Fig 2:**
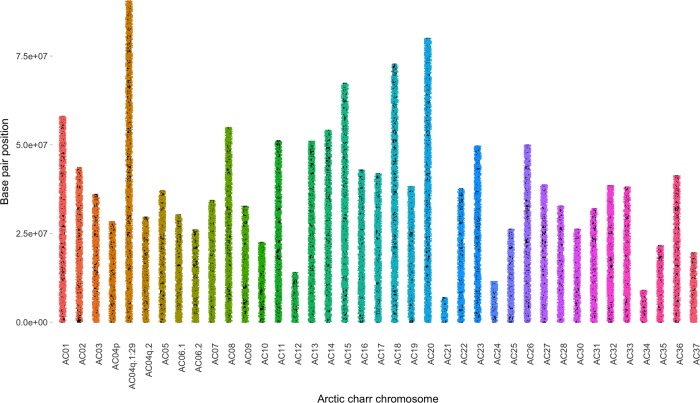
Visual representation of the distribution of the array markers among the 39 chromosomes of the Arctic charr genome (GCA_002910315.2). The black bars in the background represent the chromosome sequence, and each dot represents the location of SNP on the Arctic charr genotyping array. A total of 58,495 SNPs from the array are located along the chromosomes, while 26,425 additional SNPs are found on the unplaced contigs.

**Fig 3:**
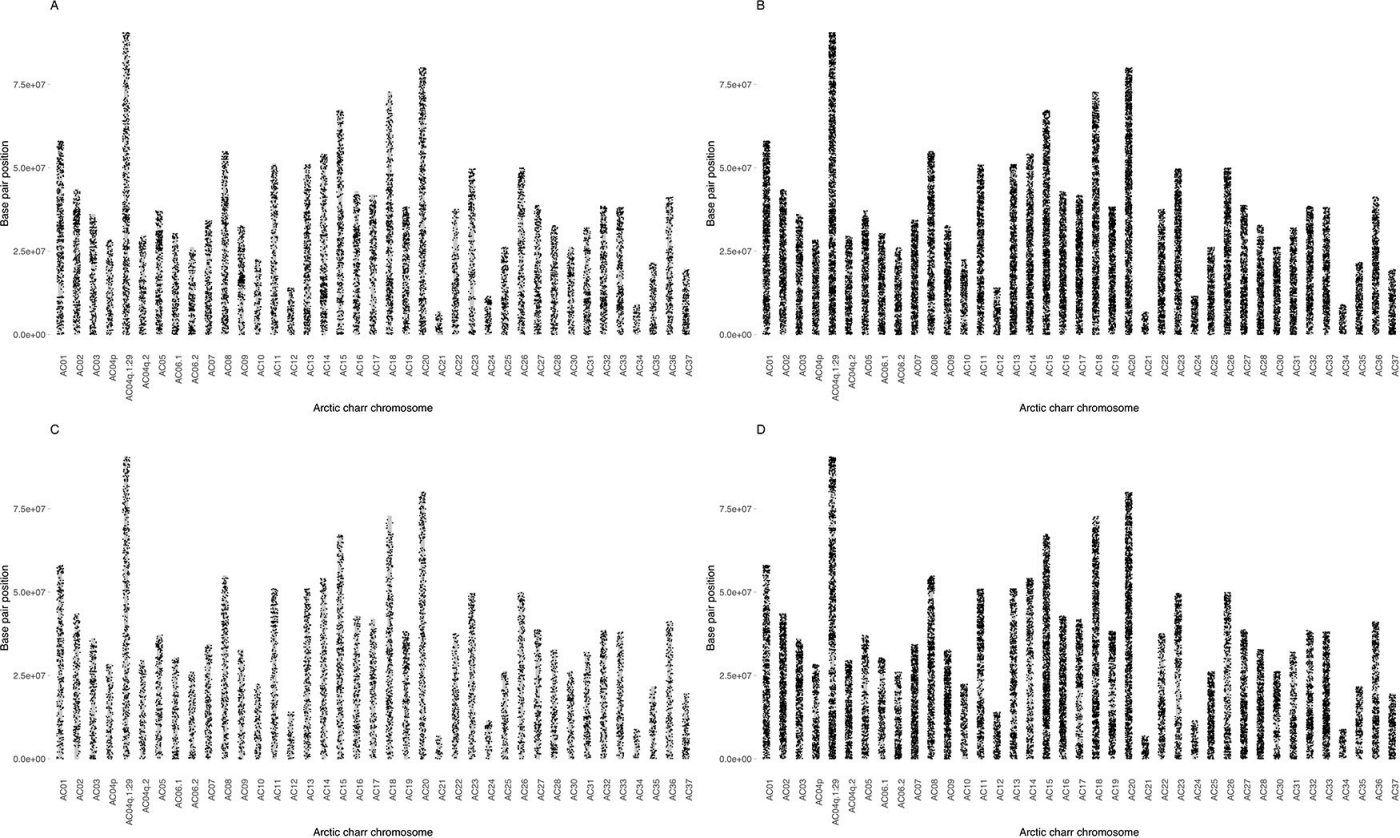
The distribution of polymorphic markers across the Arctic charr genome identified in four test groups. A: Fraser strain, Panel B: Nauyuk strain, Panel C: Tree River strain, Panel D: Icelandic.

Of the 86,503 markers on the array, 84,920 (98.2%) were successfully positioned to a single location on the new Arctic charr reference genome assembly (GenBank assembly accession: GCA_002910315.2, S2 File). 58,495 of these were distributed across the 39 chromosomes (Table 4), for an average of 38.5 markers per megabase of chromosome sequence (Fig 2, Table 4). When chromosomes were partitioned into 1Mb segments for subsequent analyses, only 3 segments on the entire genome did not contain a marker on the array. The three 1Mb segments of chromosome with no SNP were: AC01 between 58-59Mb, AC03 between 36–37 Mb, and AC06.2 between 26-27Mb. Across the whole test set, a polymorphic marker was observed every 34Kb of chromosome sequence. The average interval between polymorphic markers was lowest in the Nauyuk strain (48Kb) and higher in the Tree River (59Kb), Fraser (109Kb) and Icelandic groups (157Kb) (Fig 3). This indicates that the array provides a genome-wide characterization of genetic variation with only a few regions on the chromosomes being underrepresented.

**Table 4:** Summary table of Arctic charr reference genome coverage (GCA_002910315.2) by markers included on the 87k Arctic charr SNP genotyping array.

Chromosome name	NCBI Accession number	Number of markers	Average base pair gap between markers (kilobase pairs)	Total chromosome length (kilobase pairs)	Average markers per megabase
**AC1**	NC_036838.1	2275	25.5	58017	**39.2**
**AC2**	NC_036839.1	1641	26.5	43539	**37.7**
**AC3**	NC_036840.1	1525	23.6	36001	**42.4**
**AC4p**	NC_036841.1	1035	27.3	28293	**36.6**
**AC4q.1:29**	NC_036842.1	3230	28.0	90519	**35.7**
**AC4q.2**	NC_036843.1	1082	27.3	29596	**36.6**
**AC5**	NC_036844.1	1414	26.2	37081	**38.1**
**AC6.1**	NC_036845.1	1351	22.4	30249	**44.7**
**AC6.2**	NC_036846.1	937	27.7	26025	**36.0**
**AC7**	NC_036847.1	1449	23.7	34303	**42.2**
**AC8**	NC_036848.1	2117	25.9	54842	**38.6**
**AC9**	NC_036849.1	1285	25.4	32654	**39.4**
**AC10**	NC_036850.1	924	24.3	22457	**41.1**
**AC11**	NC_036851.1	1768	28.9	51124	**34.6**
**AC12**	NC_036852.1	468	29.8	13981	**33.5**
**AC13**	NC_036853.1	1923	26.5	50975	**37.7**
**AC14**	NC_036854.1	1961	27.6	54096	**36.3**
**AC15**	NC_036855.1	2682	25.1	67329	**39.8**
**AC16**	NC_036856.1	1623	26.4	42871	**37.9**
**AC17**	NC_036857.1	1721	24.3	41841	**41.1**
**AC18**	NC_036858.1	2617	27.8	72741	**36.0**
**AC19**	NC_036859.1	1692	22.6	38229	**44.3**
**AC20**	NC_036860.1	3350	23.9	79996	**41.9**
**AC21**	NC_036861.1	292	23.6	6905	**42.3**
**AC22**	NC_036862.1	1445	26.0	37604	**38.4**
**AC23**	NC_036863.1	1814	27.3	49633	**36.5**
**AC24**	NC_036864.1	443	25.7	11433	**38.7**
**AC25**	NC_036865.1	962	27.2	26198	**36.7**
**AC26**	NC_036866.1	1943	25.7	49931	**38.9**
**AC27**	NC_036867.1	1491	26.0	38733	**38.5**
**AC28**	NC_036868.1	1213	27.0	32734	**37.1**
**AC30**	NC_036869.1	1029	25.4	26194	**39.3**
**AC31**	NC_036870.1	1351	23.7	32007	**42.2**
**AC32**	NC_036871.1	1594	24.1	38481	**41.4**
**AC33**	NC_036872.1	1503	25.3	38085	**39.5**
**AC34**	NC_036873.1	311	28.7	8959	**34.7**
**AC35**	NC_036874.1	909	23.7	21596	**42.1**
**AC36**	NC_036875.1	1327	31.0	41233	**32.2**
**AC37**	NC_036876.1	798	24.5	19547	**40.8**

The 26,425 markers from the array not located on the chromosomes were distributed across 15,216 unplaced contigs. Of these unplaced contigs, 55.7% (8,471) contained one or more array SNPs, while 44.3% (6,744) were not represented by any SNPs on the array. The 55.7% of unplaced contigs represented by one or more SNP on the genotyping array comprise 91.6% of the sequence data within the unplaced contigs (598.5Mb out of 653.5Mb total) indicating that the smallest unplaced contigs were not well represented (S2 File).

The number of polymorphisms observed in the genome’s 15,216 unplaced contigs was sparser than within the 39 chromosomes. The percentage of contigs that had one or more polymorphic loci varied among strains (Icelandic—2,721 contigs, 17.9%; Fraser– 3,090 contigs, 20.3%; Tree River– 4,975 contigs, 32.7% and Nauyuk– 5,468 contigs, 35.9%). Thus, genetic diversity across these unplaced regions was not as well represented as across the chromosomes. This is likely in part due to the small size of these contigs relative to the chromosomes (chromosome N50: 1.02Mb, contig N50: 55.6Kb) [17]. Future efforts should focus on incorporating these contigs into the chromosomes so that they can be placed in the proper genomic context and better represented in future analyses of the genome.

### Distribution of SNPs within genes

Of the 42,439 gene entries, 22,433 genes had one or more array SNP present between their start and end positions. This indicates that 52.8% of the genes in the genome were directly represented by a SNP on the array, with between 15% and 47% of these genes possessing a polymorphic SNP among the four test groups (S3 File). This relatively sparse coverage of the genes is partially the result of the annotated genome not being available at the time of array design. The Blastn alignment of the SNP sequences to the transcriptome provided some information on which SNPs could be used to represent genes, but a SNP representative for each gene (which also passed all Affymetrix quality control metrics) could not always be identified. Even though not all genes are directly represented by a SNP on the array, the overall coverage of the genome (average of 38.5 markers per megabase of chromosome sequence) and known locations of SNPs does provide a means of associating genes of interest with nearby segregating markers.

### Sex determination

The genotypes for the 6 SNPs present in the *sdY* gene accurately predicted sex for all 463 individuals with known phenotypic sex (S3 File). The Salp87k array can therefore be used to accurately determine the sex of individuals without the need for conducting a separate analysis to genotype individuals for the *sdY* gene [55]. Sexing fish with the new array is not intended to be a direct replacement for the established method [55], which costs considerably less and is much faster. The major benefit of including the *sdY* markers on the array is that sex can be determined routinely while performing other analyses. Importantly, the *sdY* markers accurately determined sex in both North American and Icelandic Arctic charr, even though the location of the *sdY* gene is not conserved across these populations [9,15,56]. Since the *sdY* markers are associated with the *sdY* gene transcript, their performance was not influenced by the translocation position of the *sdY* gene in the Arctic charr genome.

## Conclusions

We have produced a new 87k Affymetrix Axiom genotyping array for Arctic charr and demonstrated the effective characterization of genetic variation across three Canadian aquaculture strains and several wild Icelandic populations. The array yields 14-46k polymorphic markers in each population, which is similar to documented performance of other generalist arrays that accommodate multiple species or divergent populations (range: 5–48% polymorphic array assays) [32,33]. This indicates that the Salp87k genotyping array is a generalist that provides lower amounts of information than specialized arrays (range: 83–93% polymorphic array assays), but information can be provided for wider variety of populations through the mixture of population specific and general SNPs [22]. Overall the array provides the ability to characterize both within and across population genetic diversity as well as genetic sex and it can be employed in analysis of the genetic basis of quantitative traits, the structure and pedigree of wild populations and the study of the evolutionary divergence of wild populations.

## Supporting information

S1 File SNP names, sequence and supporting information for all markers on the 87K Arctic charr genotyping array. (TSV)

S2 File Variant call format (.vcf) file containing the base pair location of all of markers on the Salp87k genotyping array that could be successfully place at a single location within the final version of the Arctic charr genome (GCA_002910315.2). Base pairs given indicate the location of the SNP, and the INFO column contains the 71mer sequence (SNP at base pair position 36) utilized in the array design.
(VCF)

S3 File Additional tables providing the breakdown in cross validation results across data sources and summary data on the distribution of polymorphic SNPs across the test set. (DOCX)
