# Supplementary material for: Design and characterization of an 87k SNP genotyping array for Arctic charr (Salvelinus alpinus)
Source: PLoS One. 2019 Apr 5;14(4):e0215008. doi: 10.1371/journal.pone.0215008 (PMC6450613; doi:10.1371/journal.pone.0215008)
Supplement: S3 File — (DOCX) [file pone.0215008.s003.docx]

**Table S1.** The cross validation of Arctic charr SNPs from different genotype by sequencing data sources. All cross validation combinations are shown, where an ‘x’ indicates population distributions.

| Fraser | Nauyuk | Icelandic families | Icelandic cave charr | Count |
| --- | --- | --- | --- | --- |
| x | **x** | **x** | **x** | 67 |
| x | **x** | **x** |  | 57 |
| x | **x** |  | **x** | 69 |
| x |  | **x** | **x** | 240 |
|  | **x** | **x** | **x** | 193 |
| x | **x** |  |  | 433 |
| x |  | **x** |  | 906 |
| x |  |  | **x** | 127 |
|  | **x** | **x** |  | 199 |
|  | **x** |  | **x** | 1307 |
|  |  | **x** | **x** | 678 |

**Table S2.** The number of shared polymorphic markers between pairs of test groups of Arctic charr. The top half of the matrix gives the total number of shared markers between two groups. And the bottom half gives the percentage of shared polymorphic markers relative to the total number of unique polymorphic markers in the union of the two groups.

|  | **Fraser** | **Nauyuk** | **Tree River** | **Icelandic** |
| --- | --- | --- | --- | --- |
| **Fraser** |  | 14,345 | 10,539 | 6,164 |
| **Nauyuk** | 28.3% |  | 26,677 | 8,137 |
| **Tree River** | 22.1% | 46.8% |  | 6,603 |
| **Icelandic** | 22.2% | 15.9% | 14.4% |  |

**Table S3.** The number of Arctic charr genes containing polymorphic SNP markers in the four test groups.

| Test group | Fraser | Nauyuk | Tree River | Wild Icelandic |
| --- | --- | --- | --- | --- |
| Genes with polymorphism | 8,854 | 20,054 | 14,867 | 6,522 |
| Genes with no polymorphism | 33,585 | 22,385 | 27,572 | 35,917 |

**Table S4.** *sdY* associated SNP genotypes in Arctic charr. Markers are all located on the partial mRNA sequence for the *sdY* gene (GenBank: JF826022.1) and display a hemizygous inheritance pattern.

| **Male phase reported for *sdY* associated SNP markers** | | | | | | | |
| --- | --- | --- | --- | --- | --- | --- | --- |
| **SNP** | **JLBFK_SDY1** | **JLBFK_SDY2** | **JLBFK_SDY3** | | **JLBFK_SDY4** | **JLBFK_SDY5** | **JLBFK_SDY6** |
| **Genotype** | BB | BB | AA | AA | | BB | AA |

| **Nucleotide residues associated with the generic alleles** | | | | | | |
| --- | --- | --- | --- | --- | --- | --- |
|  | **JLBFK_SDY1** | **JLBFK_SDY2** | **JLBFK_SDY3** | **JLBFK_SDY4** | **JLBFK_SDY5** | **JLBFK_SDY6** |
| **Allele A** | A | T | T | T | T | T |
| **Allele B** | C | G | C | C | G | C |
